# What Is the Support for Conspiracy Beliefs About COVID-19 Vaccines in Latin America? A Prospective Exploratory Study in 13 Countries

**DOI:** 10.3389/fpsyg.2022.855713

**Published:** 2022-05-06

**Authors:** Tomás Caycho-Rodríguez, José Ventura-León, Pablo D. Valencia, Lindsey W. Vilca, Carlos Carbajal-León, Mario Reyes-Bossio, Michael White, Claudio Rojas-Jara, Roberto Polanco-Carrasco, Miguel Gallegos, Mauricio Cervigni, Pablo Martino, Diego Alejandro Palacios, Rodrigo Moreta-Herrera, Antonio Samaniego-Pinho, Marlon Elías Lobos Rivera, Andrés Buschiazzo Figares, Diana Ximena Puerta-Cortés, Ibraín Enrique Corrales-Reyes, Raymundo Calderón, Bismarck Pinto Tapia, Walter L. Arias Gallegos, Olimpia Petzold

**Affiliations:** ^1^Facultad de Ciencias de la Salud, Universidad Privada del Norte, Lima, Peru; ^2^Facultad de Estudios Superiores Iztacala, Universidad Nacional Autónoma de Mexico, Tlalnepantla de Baz, Mexico; ^3^South American Center for Education and Research in Public Health, Universidad Norbert Wiener, Lima, Peru; ^4^Facultad de Psicología, Universidad Peruana de Ciencias Aplicadas, Lima, Peru; ^5^Facultad de Ciencias Humanas y Educación, Universidad Peruana Unión, Lima, Peru; ^6^Facultad de Ciencias de la Salud, Departamento de Psicología, Universidad Católica del Maule, Talca, Chile; ^7^Cuadernos de Neuropsicología, Rancagua, Chile; ^8^Programa de Pós-Graduação em Psicología, Pontificia Universidade Católica de Minas Gerais, Belo Horizonte, Brazil; ^9^Consejo Nacional de Investigaciones Científicas y Técnicas, Buenos Aires, Argentina; ^10^Facultad de Psicología, Universidad Nacional de Rosario, Rosario, Argentina; ^11^Centro Interdisciplinario de Investigaciones en Ciencias de la Salud y del Comportamiento, Universidad Adventista del Plata, Consejo Nacional de Investigaciones Científicas y Técnicas, Rosario, Argentina; ^12^Centro de Desarrollo Humano, Universidad Mariano Gálvez, Guatemala, Guatemala; ^13^Escuela de Psicología, Pontificia Universidad Católica del Ecuador, Ambato, Ecuador; ^14^Carrera de Psicología, Facultad de Filosofía, Universidad Nacional de Asunción, Asunción, Paraguay; ^15^Escuela de Psicología, Facultad de Ciencias Sociales, Universidad Tecnológica de El Salvador, San Salvador, El Salvador; ^16^Instituto Alfred Adler Uruguay, Centro de Estudios Adlerianos, Montevideo, Uruguay; ^17^Programa de Psicología, Universidad de Ibagué, Ibagué, Colombia; ^18^Servicio de Cirugía Maxilofacial, Hospital General Universitario Carlos Manuel de Céspedes, Universidad de Ciencias Médicas de Granma, Bayamo, Cuba; ^19^Carrera de Psicología, Facultad de Ciencias de la Salud, Universidad del Valle de Mexico, Mexico City, Mexico; ^20^Carrera de Psicología, Universidad Católica Boliviana San Pablo, La Paz, Bolivia; ^21^Departamento de Psicología, Universidad Católica San Pablo, Arequipa, Peru; ^22^Lone Star College-Conroe Center, Conroe, TX, United States; ^23^Psychosomatic and Psycho-Oncological Research Unit, Université Libre de Bruxelles, Brussels, Belgium

**Keywords:** beliefs, conspiracy, COVID-19, vaccine, Latin America

## Abstract

Conspiracy theories about COVID-19 began to emerge immediately after the first news about the disease and threaten to prolong the negative impact of the COVID-19 pandemic by limiting people’s willingness of receiving a life-saving vaccine. In this context, this study aimed to explore the variation of conspiracy beliefs regarding COVID-19 and the vaccine against it in 5779 people living in 13 Latin American countries (Argentina, Bolivia, Chile, Colombia, Cuba, Ecuador, El Salvador, Guatemala, Mexico, Paraguay, Peru, Uruguay and Venezuela) according to sociodemographic variables such as gender, age, educational level and source of information about COVID-19. The study was conducted during the COVID-19 pandemic between September 15 and October 25, 2021. The Spanish-language COVID-19 Vaccine Conspiracy Beliefs Scale (ECCV-COVID) and a sociodemographic survey were used. The results indicate that, in most countries, women, people with a lower educational level and those who receive information about the vaccine and COVID-19 from family/friends are more supportive of conspiracy ideas regarding the COVID-19 vaccine. In the case of age, the results vary by country. The analysis of the responses to each of the questions of the ECCV-COVID reveals that, in general, the countries evaluated are mostly in some degree of disagreement or indecision regarding conspiratorial beliefs about COVID-19 vaccines. The findings could help open further study which could support prevention and treatment efforts during the COVID-19 pandemic.

## Introduction

Since the end of 2019, the COVID-19 pandemic has become the most serious public health problem of the 21st century that has affected every country in the world (Xiao and Torok, 2020). In this regard, the control of COVID-19 depends on the effective acceptance of vaccines against the disease (Chou and Budenz, 2020). According to Our World in Data (2021), as of December 16, 2021, 56.6% of the world’s population received at least one dose of COVID-19 vaccine, 8.63 billion doses were administered, and currently, 37.22 million vaccines are administered per day; however, only 7.6% of people living in low-income countries have received at least one dose. While it generally takes approximately 10 years to develop an effective vaccine, in the case of COVID-19, 10 vaccines have been developed and tested in clinical trials since June 2020 and in December 2020, two were licensed for emergency use (Mullard, 2020).

Despite the success in the development of vaccines against COVID-19, convincing people to accept them is still a public health challenge (Al-Amer et al., 2021). Acceptance of vaccination by the general population is one of the most important factors for the success of immunization programs (DeRoo et al., 2020). In several countries, rejection and hesitancy about COVID19 vaccines are still widespread (Yang et al., 2021). A review study indicated that the acceptance of COVID-19 vaccination was over 70% in the general population, where the highest acceptance rates were found in Ecuador (97.0%), Malaysia (94.3%), Indonesia (93.3%), and China (91.3%); whereas the lowest rates were found in Kuwait (23.6%), Jordan (28.4%), Italy (53.7), Russia (54.9%), Poland (56.3%), the United States (56.9%), and France (58.9%) (Sallam, 2021). In contrast to developed countries, refusal or hesitation to accept vaccination is more common in developing countries (Arshad et al., 2021). In this regard, in Latin America, a study in six countries (Argentina, Brazil, Chile, Colombia, Mexico, and Peru) indicated that only 59% of respondents would accept a COVID-19 vaccine (Argote et al., 2021). In another study conducted in Latin America and the Caribbean, although 80% intended to be vaccinated, 81.2% also feared adverse effects (Urrunaga-Pastor et al., 2021). While these results are initial and may vary as the pandemic and vaccination processes progress, refusal or hesitation to be vaccinated against COVID-19 may jeopardize herd immunity, which would substantially limit the spread of COVID-19 (Randolph and Barreiro, 2020).

Latin American citizens tend to be less informed about public health issues (Guzman-Holst et al., 2020) and have less trust in science (Argote et al., 2021). This could be contributing to one of the main difficulties faced by vaccination programs in Latin America, which is vaccine hesitancy due to conspiracy beliefs. The emergence of conspiracy beliefs may also be associated with unnecessarily alarming and sensationalist media reports (Rovetta, 2021). Conspiracy theories about COVID-19 began to emerge immediately after the first news about the disease (Douglas, 2021) and threaten to prolong the negative impact of the COVID-19 pandemic by limiting people’s willingness to receive a vaccine that could save their lives (Jensen et al., 2021). Conspiracy beliefs are attempts to explain social and political events or situations on the basis of ideas of secret plots led by two or more powerful actors (Douglas et al., 2019). These types of beliefs usually appear in situations of social crisis, which generate greater uncertainty and collective fear (van Prooijen and Douglas, 2017), and which are responses to psychological needs to try to understand complex threatening situations in a simple and predictable way (Franks et al., 2013; Douglas et al., 2017). In this sense, it is not surprising that conspiracy beliefs emerged during the COVID-19 pandemic and that misinformation about the disease and vaccines spread rapidly (Kouzy et al., 2020). This phenomenon was also observed during the Spanish flu pandemic (Spinney, 2017) and the H1N1 outbreak (Bangerter et al., 2012).

Conspiracy beliefs related to the COVID-19 vaccine have negatively affected intentions to be vaccinated against COVID-19 (Bertin et al., 2020; Freeman et al., 2020a), to a much greater extent than belief in more general theories about COVID-19 (Yang et al., 2021). With the development of COVID-19 vaccines, different conspiracy theories have been proposed, where the most widely accepted ones refer to the installation of 5G chips in people, the generation of infertility, or death from inoculation with the COVID-19 vaccine (Chou and Budenz, 2020; Romer and Jamieson, 2020; Uscinski et al., 2020). People who believe in conspiracies tend to resist preventive measures and vaccination proposed by scientists or health experts (Douglas et al., 2017, 2019). Likewise, belief in conspiracy theories can trigger negative public emotions, which generate vaccine hesitancy and decreased vaccine acceptance (Yang et al., 2021).

Different studies have shown that people with greater scientific knowledge about a topic were less likely to believe in these conspiracy theories and thus reduce negative consequences on vaccine adoption (Swami et al., 2014; Sallam et al., 2020a; Yang et al., 2021). Likewise, conspiracy beliefs lead to the rejection of, or hesitancy in receiving, vaccines, due to the fact that they generate distrust in governments, health care institutions and the pharmaceutical industry (Bertin et al., 2020; Hornsey et al., 2020). Likewise, there are different sociodemographic variables that are associated in some way with conspiracy beliefs. Thus, it has been suggested that approximately 30% of people between 30 and 39 years of age agreed with conspiracy ideas, such as that the pandemic is a global effort to force everyone to comply with mandatory vaccination, while only 8% of those older than 80 agreed with this type of beliefs; however, gender does not seem to play an important role in conspiracy ideas, which only explained 3% of the variation in conspiracy beliefs (Jensen et al., 2021). Similarly, because social networks are the main source of dissemination of conspiracy beliefs, users of this information medium are more likely to believe in these ideas (Arshad et al., 2021; Suarez-Lledo and Alvarez-Galvez, 2021). Another study differed to a degree by reported that women, people with lower educational levels, and those who relied on social networking platforms as the main source of information presented higher conspiracy beliefs about COVID-19 vaccines (Romer and Jamieson, 2020; Sallam et al., 2021a).

Despite the number of studies which have established negative correlations between conspiracy belief and intentions to be vaccinated before and during the COVID-19 pandemic, the effect size remains moderate (Jolley and Douglas, 2014; Bertin et al., 2020; Roozenbeek et al., 2020; Salali and Uysal, 2020). Therefore, the variation in conspiracy beliefs about the COVID-19 vaccine among different countries needs to be explained. Furthermore, a review of the current scientific literature indicated that the topic has not been sufficiently investigated in a large sample of Latin American countries. It is important to fill this knowledge gap, even more so at a time when conspiracy beliefs are openly discussed by the general population (Jensen et al., 2021). Also, this study will provide further information to elucidate the variation in conspiracy beliefs about the COVID-19 vaccine according to certain sociodemographic variables, given that previous findings are sometimes contradictory (Eberhardt and Ling, 2021). In this context, the current study aimed to explore the variation of conspiracy beliefs against COVID-19 vaccines in a group of people residing in 13 Latin American countries according to sociodemographic variables such as gender, age, educational level, and source of information about COVID-19. The findings obtained in this study could contribute to effectively combat the dissemination of erroneous information about the vaccines, design strategies to generate confidence in the general population, and increase the acceptance rate of the vaccine against COVID-19.

## Materials and Methods

### Participants

A total of 5779 people residing in 13 Spanish-speaking Latin American countries (Argentina, Bolivia, Chile, Colombia, Cuba, Ecuador, El Salvador, Guatemala, Mexico, Paraguay, Peru, Uruguay, and Venezuela) participated in the study, selected through non-probability snowball sampling, where each respondent was encouraged to invite family and friends to participate in the study (Naderifar et al., 2017). It has been suggested that the use of this type of sampling in mental health surveys during the current pandemic might introduce some type of bias that is difficult to control for Pierce et al. (2020). However, snowball sampling through social networks has proven to be an effective and rapid strategy to engage a larger number of people (Baltar and Brunet, 2012). In addition, due to social interaction limitations during the pandemic, which did not allow for in-person data collection, snowball sampling was an appropriate way to reach participants. Recent studies during the COVID-19 pandemic have also successfully used this type of sampling in multinational studies (for example, Öcal et al., 2020; Kolakowsky-Hayner et al., 2021) as well as in studies referring to conspiracy beliefs about the pandemic (such as Khokhlova et al., 2021).

All participants had to be of legal age and give informed consent to participate in the study. The number of participants in each country varied between 322 (Peru) and 746 (El Salvador). A total of 4092 women and 1687 men participated, with a mean age of 33.28 years old (SD = 13.48), with the Mexican sample being the youngest (*M* = 24.66, SD = 8.65) and the Guatemalan sample having the highest mean age (*M* = 44.04, SD = 13.62). In addition, 4893 participants had higher education (84.67%) and 1871 (32.38%) reported that their main source of information about the COVID-19 vaccine was social networks (Facebook, Instagram or others). Table 1 shows, in more detail, the sociodemographic information for each country.

**TABLE 1:** Sociodemographic information of the participants.

Variables/ Countries	Argentina	Bolivia	Chile	Colombia	Cuba	Ecuador	El Salvador	Guatemala	Mexico	Paraguay	Peru	Uruguay	Venezuela
	(*n* = 363)	(*n* = 564)	(*n* = 453)	(*n* = 461)	(*n* = 334)	(*n* = 438)	(*n* = 746)	(*n* = 420)	(*n* = 484)	(*n* = 417)	(*n* = 322)	(*n* = 392)	(*n* = 385)
**Gender (%)**													
Female	255 (70.25)	421 (74.65)	314 (69.32)	322 (69.85)	231 (69.16)	311 (71)	546 (73.19)	297 (70.71)	331 (68.39)	292 (70.02)	224 (69.57)	272 (69.39)	276 (71.69)
Male	108 (29.75)	143 (25.35)	139 (30.68)	139 (30.15)	103 (30.84)	127 (29)	200 (26.81)	123 (29.29)	153 (31.61)	125 (29.98)	98 (30.43)	120 (30.61)	109 (28.31)
**Age (%)**													
<23	54 (14.88)	31 (5.5)	60 (13.25)	266 (57.7)	143 (42.81)	146 (33.33)	170 (22.79)	14 (3.33)	282 (58.26)	30 (7.19)	112 (34.78)	40 (10.2)	66 (17.14)
23–42	192 (52.89)	333 (59.04)	267 (58.94)	125 (27.11)	156 (46.71)	227 (51.83)	426 (57.1)	198 (47.14)	171 (35.33)	323 (77.46)	191 (59.32)	264 (67.35)	92 (23.9)
>42	117 (32.23)	200 (35.46)	126 (27.81)	70 (15.18)	35 (10.48)	65 (14.84)	150 (20.11)	208 (49.52)	31 (6.4)	64 (15.35)	19 (5.9)	88 (22.45)	227 (58.96)
**Highest level of education (%)**													
Primary	48 (13.22)	19 (3.37)	32 (7.06)	159 (34.49)	6 (1.8)	93 (21.23)	282 (37.8)	36 (8.57)	44 (9.09)	28 (6.71)	46 (14.29)	60 (15.31)	33 (8.57)
University	315 (86.78)	545 (96.63)	421 (92.94)	302 (65.51)	328 (98.2)	345 (78.77)	464 (62.2)	384 (91.43)	440 (90.91)	389 (93.29)	276 (85.71)	332 (84.69)	352 (91.43)
**Sources of information (%)**													
Government,	105 (28.93)	79 (14.01)	165 (36.42)	104 (22.56)	104 (31.14)	144 (32.88)	262 (35.12)	110 (26.19)	183 (37.81)	176 (42.21)	115 (35.71)	148 (37.76)	36 (9.35)
Family/friends, etc.	19 (5.23)	24 (4.26)	20 (4.42)	58 (12.58)	27 (8.08)	34 (7.76)	43 (5.76)	43 (10.24)	50 (10.33)	21 (5.04)	18 (5.59)	37 (9.44)	42 (10.91)
Social networks	77 (21.21)	214 (37.94)	137 (30.24)	149 (32.32)	51 (15.27)	146 (33.33)	298 (39.95)	147 (35)	143 (29.55)	118 (28.3)	93 (28.88)	69 (17.6)	229 (59.48)
Television, radio and newspapers	162 (44.63)	247 (43.79)	131 (28.92)	150 (32.54)	152 (45.51)	114 (26.03)	143 (19.17)	120 (28.57)	108 (22.31)	102 (24.46)	96 (29.81)	138 (35.2)	78 (20.26)

### Instruments

#### Sociodemographic Variables

Participants completed initial sociodemographic questions, which included information on gender (binary variable: male and female), age (three categories: <23 years old, 23 to 42 years old, >42 years old), which was recoded into quartiles to summarize the large amount of age-related data, educational level (binary variable: basic studies and higher education), and sources of information about COVID-19 (four categories: television, radio, and print media; official government sources; social networks; family members/friends).

#### Conspiracy Beliefs About COVID-19 Vaccines

The Vaccine Conspiracy Beliefs Scale-COVID-19 (VCBS-COVID-19; Caycho-Rodríguez et al., 2022) was used. The ECCV-COVID was developed from the Vaccine Conspiracy Beliefs Scale (VCBS; Shapiro et al., 2016) and assesses conspiratorial thinking about COVID-19 immunizations through 7 items. Respondents indicate how much they agree or disagree with each item on a scale of 7 response alternatives ranging from “strongly disagree” (1) to “strongly agree” (7).

**FIGURE 1:**
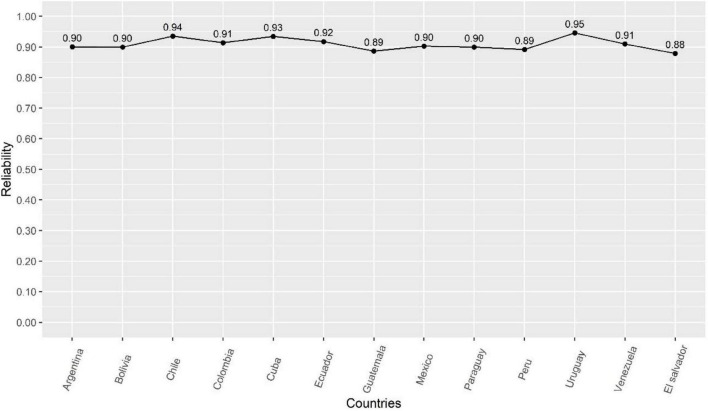
Reliability of the ECCV-COVID in the 13 Latin American countries.

For the development of the ECCV-COVID, the original VCBS was first translated using the back-translation method. Second, two independent investigators, one a subject matter specialist familiar with COVID-19 vaccination and bilingual in English and Spanish, and the other an English language specialist, translated the VCBS from English to Spanish. Subsequently, two other investigators, one a subject matter expert and the other a language expert, who were not familiar with the first translation, translated the Spanish version back into English. Then, both versions were compared looking for possible inconsistencies in order to generate a harmonized version. An example of the items is: “Vaccine safety information is often made up.” To assess conspiracy beliefs about COVID-19 vaccines, the term “COVID-19” was added to each of the VCBS items. For example, “Information about the safety of COVID-19 vaccines is often made up.” The ECCV-COVID has been shown to be unidimensional, reliable (with alpha and omega coefficient values ranging from 0.87 to 0.94) and invariant across 13 Latin American countries (Caycho-Rodríguez et al., 2022). The reliability of the ECCV-COVID for each country is shown in Figure 1. The total score of the ECCV-COVID ranges from 7 to 49, where higher values indicate a higher degree of agreement with conspiracy beliefs. The ECCV-COVID can be found in Appendix 1.

### Procedure

**FIGURE 2:**
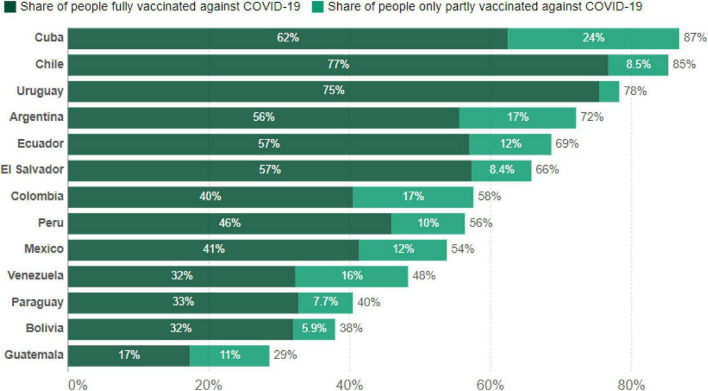
Percentage of people vaccinated against COVID-19 by October 25, 2021 in participating countries based on data derived from Our World in Data (2021); https://ourworldindata.org.

The study was part of a larger project and was conducted during the COVID-19 pandemic between September 15 and October 25, 2021. During this time period, between 29 and 87% of people residing in the countries evaluated were fully or partially vaccinated against COVID-19. According to Figure 2, Chile (77%) and Uruguay (75%) had the highest proportion of people fully vaccinated against COVID-19, while Guatemala had the lowest proportion of people fully or partially vaccinated (17%).

Data were collected simultaneously in the 13 participating countries and the collection procedure was the same in each country. An online questionnaire was created using Google Forms, which was distributed by email and on different social media platforms (Facebook, Instagram, and WhatsApp). The online questionnaire included questions related to sociodemographic data, conspiracy beliefs about COVID-19 vaccines, and other associated variables. The online survey allows for easy data collection, maintains respondent anonymity, reduces bias, and helps to obtain complete responses as participants answer all required questions (Andrews et al., 2003). Finally, online surveys allow participants’ responses to be saved directly to a file, reducing the work of data entry and thus avoiding transcription errors (Evans and Mathur, 2005). Participants completed the online survey in approximately 10 min. Participation in the study was voluntary, participants gave informed consent after reading the study objectives before continuing with the survey, and no financial compensation was received for participation. Participants were asked to answer all questions in the questionnaire before submitting their responses. The study was approved by the Ethics Committee of the Universidad Privada del Norte in Peru (registration number: 20213002).

### Data Analysis

Data analysis was performed with the R programming language in its RStudio environment. The libraries used were ‘ggplot2’ version 3.3.5 (Wickham et al., 2020) for plotting, ‘tidyverse’ version 1.1.4 (Wickham, 2019) for organizing and estimating statistics and ‘effectsize’ version 0.6.0.1 (Ben-Shachar et al., 2020) for calculating effect sizes.

**FIGURE 3:**
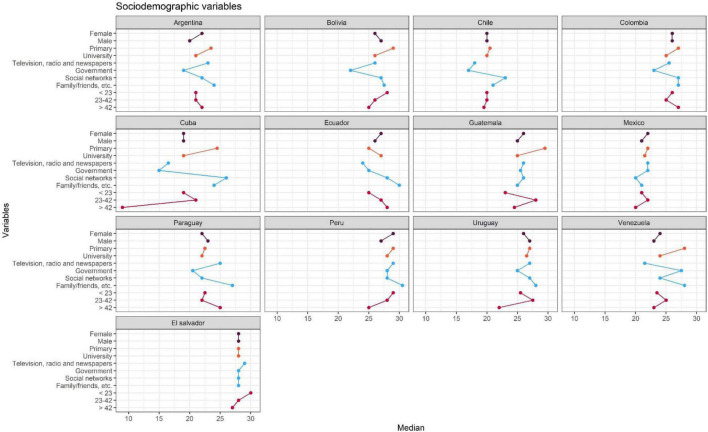
Median for each of the sociodemographic variables by country.

Given that the presence of outliers was preliminarily verified through the box plot (see Supplementary Figure 1), we opted to use the median, which is robust in handling outliers, and the interquartile ranges, which are by antonomasia its measure of dispersion. The median was calculated by country and comparison variable (gender, age ranges, educational levels, COVID-19 information sources) and displayed in a dot and line graph (Figure 3), which allows for a quick visualization of the conspiracy scores. For interpretation, the position of the point (median) should be considered. Points positioned to the right indicate a higher degree of support for conspiratorial ideas, while points positioned to the left indicate a lower degree. It is important to note that statistical significance tests (*p*-value, α) or probabilistic models (Shapiro-Wilk, Q-Q plots) are not used in this study for two reasons: (a) it requires random sampling (Hirschauer et al., 2020) and the present study used non-probability convenience sampling which is usual in psychology (Kline, 2015) and (b) when there is a lot of data (*n* = 5779) these models are sensitive to reject the null hypothesis (Lin et al., 2013). In this sense, this study has a descriptive rather than inferential intent. This does not detract from the importance, but rather informs the scope of the research and limitations in the external validity of the study.

Since the presence of outliers was found, the ordinary Cohen’s d was not used as a measure of comparison (Rousselet et al., 2017), but rather a robust version (δ), which has as its central characteristic that it works quite well in unequal sample sizes (Wilcox and Tian, 2011) and unequal variances (Algina et al., 2005). Its interpretation is similar to its standard version where: δ: ≥0.30, small; δ: ≥0.50, medium; δ: ≥0.80 is large (Cohen, 1988). In the case of variables with more than two categories (age range, COVID-19 information sources) explanatory measure of effect size (ξ) was used, which also presents robustness for variance inequality and groups (Wilcox, 2017). Its interpretation is that 0.10, 0.30, and 0.50 correspond to small, medium and large effect sizes (Mair and Wilcox, 2020).

## Results

Table 2 presents the arithmetic means and standard deviations grouped by country and each of the comparison variables (gender, age ranges, educational levels, sources of COVID-19 information). It is worth noting that, in most countries, women, people with lower educational levels, those who receive information about COVID-19 and the vaccines from family and friends are those people who are more supportive of conspiracy ideas against the COVID-19 vaccine. In the case of age, the results vary greatly. However, Cuba and Venezuela present noteworthy variations. In addition, Figure 3 summarizes this information visually and gives an overview of the results, where the points indicate the value of the median obtained in that country and in the comparison group. In relation to the effect sizes, in most countries the differences between the comparison variables that can be attributed to the scores of the conspiracy scale are minimal; although it is worth highlighting the variations in the sources of information in countries such as Cuba (ξ = 0.43) and Ecuador (ξ = 0.31) as well as age ranges (ξ = 0.43) that occurred in Cuba.

**TABLE 2:** Descriptive statistics and effect size.

Variables/Countries	Argentina	Bolivia	Chile	Colombia	Cuba	Ecuador	El salvador	Guatemala	Mexico	Paraguay	Peru	Uruguay	Venezuela
**Gender [Md (IQR)]**													
Female	14 (22)	12 (26)	16 (20)	14 (26)	15 (19)	16 (27)	12 (28)	12 (26)	15 (22)	14.25 (22)	11 (29)	15 (26)	15 (24)
Male	13 (20)	14 (27)	17 (20)	14 (26)	16 (19)	14 (26)	12 (28)	13 (25)	15 (21)	11 (23)	11 (27)	17 (27)	15 (23)
δ	−0.18	0.04	−0.03	0.00	0.00	−0.03	0.04	−0.10	0.09	−0.24	0.03	0.03	−0.05
**Age [Md (IQR)]**													
<23	21 (15)	28 (13)	20 (16.25)	26 (13)	19 (13)	25 (14)	30 (11)	23 (7)	21 (15)	22.5 (13)	29 (10)	25.5 (13.25)	23.5 (14)
23–42	21 (13)	26 (12)	20 (16)	25 (14)	21 (15.25)	27 (14)	28 (12)	28 (13)	22 (16)	22 (14)	28 (11)	27.5 (15)	25 (15.25)
>42	15 (22)	13 (25)	16.25 (19.5)	13 (27)	13 (9)	14 (28)	11 (27)	7 (24.5)	15 (20)	13 (25)	10 (25)	13.25 (22)	14 (23)
ξ	0.10	0.20	0.06	0.08	0.46	0.09	0.21	0.16	0.13	0.26	0.16	0.10	0.18
**Highest level of education [Md (IQR)]**													
Primary	23.5 (13)	29 (12)	20.5 (17)	27 (12)	24.5 (20)	25 (13)	28 (11)	29.5 (10.5)	22 (17.5)	22.5 (15.75)	29 (9)	27 (15.5)	28 (8)
University	21 (14)	26 (12)	20 (16)	25 (14)	19 (15)	27 (15)	28 (12)	25 (13)	21.5 (15)	22 (14)	28 (12)	26.5 (15)	24 (15)
δ	0.07	0.27	0.05	0.13	0.24	0.07	0.27	0.06	0.06	0.10	0.06	0.21	0.02
**Sources of information [Md (IQR)]**													
Government,	19 (14)	22 (19)	17 (14)	23 (14)	15 (15.25)	25 (14.5)	28 (14)	25.5 (15)	22 (15)	20.5 (14)	28 (12)	25 (16)	27.5 (21.5)
Family/friends, etc.	24 (10)	27.5 (5.25)	2 (15.25)	27 (12)	24 (12)	30 (11)	28 (20)	25 (12)	21 (16)	27 (10)	30.5 (10)	28 (17)	28 (18)
Social networks,	22 (13)	27 (13)	23 (15)	27 (15)	26 (18)	28 (14)	28 (11)	26 (12)	20 (16)	22 (13)	28 (13)	27 (14)	24 (14)
Television, radio and newspapers	23 (13)	26 (12)	18 (16)	25.5 (14)	16.5 (13)	24 (15)	29 (10)	26 (13.25)	22 (16)	25 (13)	29 (8)	27 (13)	21.5 (17)
ξ	0.29	0.20	0.19	0.14	0.43	0.31	0.09	0.10	0.28	0.17	0.20	0.19	0.10

Table 3 shows the response rates for each of the ECCV items by country. For each item, the categories with the highest response rates are 1 (“*Strongly Disagree*”) and 4 (“*Neutral*”). In order to decide which countries have more of these alternatives, a cut-off of greater than 30% was established in some of them. Thus, Chile, Cuba, Mexico, and Argentina have the highest response rates in alternative 1 and El Salvador, Peru and Colombia in alternative 4 in almost all the items; with the exception of item 2 (“*Vaccinating children against COVID-19 is harmful and this fact is hidden.* “) where there is a higher percentage of both response alternatives (1 and 4) in 9 out of 13 countries. Specifically, Cuba and Argentina show response rates higher than 40% for alternative 1. Likewise, vcbs2 presents the largest effect size (ξ^2^ = 0.31).

**TABLE 3:** Response rates for each item and by country.

Items/countries	*Strongly Disagree*	*Disagree*	*Somewhat Disagree*	*Neutral*	*Somewhat Agree*	*Agree*	*Strongly Agree*	ξ
**Item 1 n (%)**								
Argentina	69 (19.01)	43 (11.85)	34 (9.37)	104 (28.65)	45 (12.4)	35 (9.64)	33 (9.09)	0.20
Bolivia	74 (13.12)	83 (14.72)	70 (12.41)	165 (29.26)	80 (14.18)	48 (8.51)	44 (7.8)	
Chile	138 (30.46)	65 (14.35)	55 (12.14)	83 (18.32)	47 (10.38)	30 (6.62)	35 (7.73)	
Colombia	77 (16.7)	34 (7.38)	47 (10.2)	148 (32.1)	69 (14.97)	37 (8.03)	49 (10.63)	
Cuba	95 (28.44)	32 (9.58)	51 (15.27)	72 (21.56)	37 (11.08)	29 (8.68)	18 (5.39)	
Ecuador	65 (14.84)	37 (8.45)	50 (11.42)	113 (25.8)	68 (15.53)	43 (9.82)	62 (14.16)	
El Salvador	104 (13.94)	57 (7.64)	67 (8.98)	216 (28.95)	108 (14.48)	89 (11.93)	105 (14.08)	
Guatemala	59 (14.05)	37 (8.81)	47 (11.19)	87 (20.71)	77 (18.33)	57 (13.57)	56 (13.33)	
Mexico	98 (20.25)	58 (11.98)	44 (9.09)	136 (28.1)	62 (12.81)	38 (7.85)	48 (9.92)	
Paraguay	81 (19.42)	53 (12.71)	57 (13.67)	85 (20.38)	53 (12.71)	38 (9.11)	50 (11.99)	
Peru	42 (13.04)	21 (6.52)	33 (10.25)	94 (29.19)	52 (16.15)	39 (12.11)	41 (12.73)	
Uruguay	57 (14.54)	43 (10.97)	55 (14.03)	105 (26.79)	40 (10.2)	35 (8.93)	57 (14.54)	
Venezuela	81 (21.04)	59 (15.32)	40 (10.39)	83 (21.56)	46 (11.95)	32 (8.31)	44 (11.43)	
**Item 2 (%)**								
Argentina	162 (44.63)	49 (13.5)	49 (13.5)	69 (19.01)	8 (2.2)	10 (2.75)	16 (4.41)	0.31
Bolivia	130 (23.05)	77 (13.65)	85 (15.07)	170 (30.14)	35 (6.21)	34 (6.03)	33 (5.85)	
Chile	178 (39.29)	78 (17.22)	59 (13.02)	84 (18.54)	17 (3.75)	13 (2.87)	24 (5.3)	
Colombia	138 (29.93)	59 (12.8)	79 (17.14)	126 (27.33)	25 (5.42)	20 (4.34)	14 (3.04)	
Cuba	207 (61.98)	27 (8.08)	47 (14.07)	37 (11.08)	5 (1.5)	4 (1.2)	7 (2.1)	
Ecuador	118 (26.94)	64 (14.61)	56 (12.79)	102 (23.29)	43 (9.82)	20 (4.57)	35 (7.99)	
El Salvador	143 (19.17)	74 (9.92)	67 (8.98)	238 (31.9)	83 (11.13)	71 (9.52)	70 (9.38)	
Guatemala	134 (31.9)	39 (9.29)	60 (14.29)	109 (25.95)	31 (7.38)	22 (5.24)	25 (5.95)	
Mexico	183 (37.81)	69 (14.26)	56 (11.57)	124 (25.62)	21 (4.34)	16 (3.31)	15 (3.1)	
Paraguay	143 (34.29)	61 (14.63)	70 (16.79)	99 (23.74)	12 (2.88)	14 (3.36)	18 (4.32)	
Peru	70 (21.74)	22 (6.83)	57 (17.7)	96 (29.81)	36 (11.18)	21 (6.52)	20 (6.21)	
Uruguay	72 (18.37)	51 (13.01)	52 (13.27)	136 (34.69)	27 (6.89)	20 (5.1)	34 (8.67)	
Venezuela	100 (25.97)	46 (11.95)	51 (13.25)	100 (25.97)	26 (6.75)	27 (7.01)	35 (9.09)	
**Item 3 n (%)**								
Argentina	81 (22.31)	62 (17.08)	48 (13.22)	101 (27.82)	24 (6.61)	26 (7.16)	21 (5.79)	0.26
Bolivia	77 (13.65)	57 (10.11)	78 (13.83)	166 (29.43)	79 (14.01)	44 (7.8)	63 (11.17)	
Chile	95 (20.97)	74 (16.34)	79 (17.44)	102 (22.52)	40 (8.83)	28 (6.18)	35 (7.73)	
Colombia	81 (17.57)	55 (11.93)	70 (15.18)	126 (27.33)	65 (14.1)	31 (6.72)	33 (7.16)	
Cuba	107 (32.04)	42 (12.57)	54 (16.17)	81 (24.25)	18 (5.39)	19 (5.69)	13 (3.89)	
Ecuador	65 (14.84)	44 (10.05)	61 (13.93)	116 (26.48)	51 (11.64)	40 (9.13)	61 (13.93)	
El Salvador	86 (11.53)	65 (8.71)	83 (11.13)	240 (32.17)	104 (13.94)	84 (11.26)	84 (11.26)	
Guatemala	62 (14.76)	46 (10.95)	55 (13.1)	112 (26.67)	60 (14.29)	34 (8.1)	51 (12.14)	
Mexico	127 (26.24)	72 (14.88)	66 (13.64)	135 (27.89)	40 (8.26)	22 (4.55)	22 (4.55)	
Paraguay	97 (23.26)	54 (12.95)	82 (19.66)	101 (24.22)	36 (8.63)	24 (5.76)	23 (5.52)	
Peru	41 (12.73)	24 (7.45)	45 (13.98)	93 (28.88)	52 (16.15)	40 (12.42)	27 (8.39)	
Uruguay	46 (11.73)	35 (8.93)	62 (15.82)	106 (27.04)	55 (14.03)	33 (8.42)	55 (14.03)	
Venezuela	66 (17.14)	52 (13.51)	53 (13.77)	88 (22.86)	40 (10.39)	40 (10.39)	46 (11.95)	
**Item 4 n (%)**								
Argentina	98 (27)	64 (17.63)	62 (17.08)	79 (21.76)	28 (7.71)	19 (5.23)	13 (3.58)	0.22
Bolivia	79 (14.01)	69 (12.23)	100 (17.73)	134 (23.76)	72 (12.77)	53 (9.4)	57 (10.11)	
Chile	136 (30.02)	87 (19.21)	71 (15.67)	75 (16.56)	35 (7.73)	27 (5.96)	22 (4.86)	
Colombia	87 (18.87)	62 (13.45)	72 (15.62)	110 (23.86)	59 (12.8)	34 (7.38)	37 (8.03)	
Cuba	111 (33.23)	46 (13.77)	56 (16.77)	62 (18.56)	17 (5.09)	29 (8.68)	13 (3.89)	
Ecuador	87 (19.86)	49 (11.19)	65 (14.84)	98 (22.37)	47 (10.73)	47 (10.73)	45 (10.27)	
El Salvador	117 (15.68)	71 (9.52)	99 (13.27)	226 (30.29)	80 (10.72)	78 (10.46)	75 (10.05)	
Guatemala	83 (19.76)	53 (12.62)	64 (15.24)	97 (23.1)	55 (13.1)	32 (7.62)	36 (8.57)	
Mexico	158 (32.64)	63 (13.02)	64 (13.22)	115 (23.76)	37 (7.64)	21 (4.34)	26 (5.37)	
Paraguay	104 (24.94)	67 (16.07)	90 (21.58)	81 (19.42)	31 (7.43)	19 (4.56)	25 (6)	
Peru	50 (15.53)	19 (5.9)	51 (15.84)	81 (25.16)	56 (17.39)	33 (10.25)	32 (9.94)	
Uruguay	63 (16.07)	57 (14.54)	56 (14.29)	107 (27.3)	33 (8.42)	31 (7.91)	45 (11.48)	
Venezuela	83 (21.56)	67 (17.4)	70 (18.18)	67 (17.4)	42 (10.91)	21 (5.45)	35 (9.09)	
**Item 5 n (%)**								
Argentina	96 (26.45)	57 (15.7)	59 (16.25)	80 (22.04)	30 (8.26)	28 (7.71)	13 (3.58)	0.21
Bolivia	80 (14.18)	91 (16.13)	93 (16.49)	154 (27.3)	63 (11.17)	42 (7.45)	41 (7.27)	
Chile	139 (30.68)	100 (22.08)	70 (15.45)	76 (16.78)	23 (5.08)	29 (6.4)	16 (3.53)	
Colombia	79 (17.14)	67 (14.53)	69 (14.97)	126 (27.33)	61 (13.23)	31 (6.72)	28 (6.07)	
Cuba	102 (30.54)	48 (14.37)	64 (19.16)	57 (17.07)	22 (6.59)	29 (8.68)	12 (3.59)	
Ecuador	81 (18.49)	48 (10.96)	71 (16.21)	106 (24.2)	48 (10.96)	37 (8.45)	47 (10.73)	
El Salvador	108 (14.48)	67 (8.98)	91 (12.2)	239 (32.04)	95 (12.73)	77 (10.32)	69 (9.25)	
Guatemala	70 (16.67)	50 (11.9)	64 (15.24)	105 (25)	50 (11.9)	34 (8.1)	47 (11.19)	
Mexico	141 (29.13)	74 (15.29)	68 (14.05)	122 (25.21)	33 (6.82)	27 (5.58)	19 (3.93)	
Paraguay	101 (24.22)	59 (14.15)	81 (19.42)	98 (23.5)	30 (7.19)	23 (5.52)	25 (6)	
Peru	43 (13.35)	19 (5.9)	55 (17.08)	85 (26.4)	59 (18.32)	42 (13.04)	19 (5.9)	
Uruguay	69 (17.6)	56 (14.29)	63 (16.07)	101 (25.77)	40 (10.2)	25 (6.38)	38 (9.69)	
Venezuela	89 (23.12)	53 (13.77)	66 (17.14)	81 (21.04)	30 (7.79)	35 (9.09)	31 (8.05)	
**Item 6 n (%)**								
Argentina	92 (25.34)	62 (17.08)	71 (19.56)	81 (22.31)	25 (6.89)	20 (5.51)	12 (3.31)	0.22
Bolivia	80 (14.18)	87 (15.43)	93 (16.49)	155 (27.48)	64 (11.35)	46 (8.16)	39 (6.91)	
Chile	138 (30.46)	86 (18.98)	68 (15.01)	73 (16.11)	41 (9.05)	23 (5.08)	24 (5.3)	
Colombia	83 (18)	67 (14.53)	71 (15.4)	123 (26.68)	54 (11.71)	33 (7.16)	30 (6.51)	
Cuba	111 (33.23)	58 (17.37)	61 (18.26)	51 (15.27)	23 (6.89)	23 (6.89)	7 (2.1)	
Ecuador	83 (18.95)	53 (12.1)	66 (15.07)	113 (25.8)	40 (9.13)	32 (7.31)	51 (11.64)	
El Salvador	114 (15.28)	66 (8.85)	91 (12.2)	230 (30.83)	99 (13.27)	72 (9.65)	74 (9.92)	
Guatemala	72 (17.14)	54 (12.86)	66 (15.71)	107 (25.48)	52 (12.38)	27 (6.43)	42 (10)	
Mexico	155 (32.02)	62 (12.81)	78 (16.12)	118 (24.38)	35 (7.23)	18 (3.72)	18 (3.72)	
Paraguay	101 (24.22)	62 (14.87)	87 (20.86)	96 (23.02)	25 (6)	18 (4.32)	28 (6.71)	
Peru	43 (13.35)	20 (6.21)	50 (15.53)	85 (26.4)	60 (18.63)	41 (12.73)	23 (7.14)	
Uruguay	66 (16.84)	55 (14.03)	55 (14.03)	93 (23.72)	44 (11.22)	34 (8.67)	45 (11.48)	
Venezuela	91 (23.64)	52 (13.51)	70 (18.18)	75 (19.48)	29 (7.53)	30 (7.79)	38 (9.87)	
**Item 7 n (%)**								
Argentina	121 (33.33)	46 (12.67)	60 (16.53)	88 (24.24)	17 (4.68)	17 (4.68)	14 (3.86)	0.22
Bolivia	98 (17.38)	79 (14.01)	73 (12.94)	167 (29.61)	64 (11.35)	43 (7.62)	40 (7.09)	
Chile	123 (27.15)	89 (19.65)	58 (12.8)	76 (16.78)	48 (10.6)	22 (4.86)	37 (8.17)	
Colombia	80 (17.35)	56 (12.15)	59 (12.8)	126 (27.33)	54 (11.71)	41 (8.89)	45 (9.76)	
Cuba	127 (38.02)	46 (13.77)	59 (17.66)	62 (18.56)	14 (4.19)	12 (3.59)	14 (4.19)	
Ecuador	78 (17.81)	45 (10.27)	61 (13.93)	122 (27.85)	49 (11.19)	37 (8.45)	46 (10.5)	
El Salvador	111 (14.88)	67 (8.98)	72 (9.65)	222 (29.76)	102 (13.67)	76 (10.19)	96 (12.87)	
Guatemala	79 (18.81)	57 (13.57)	65 (15.48)	112 (26.67)	38 (9.05)	25 (5.95)	44 (10.48)	
Mexico	140 (28.93)	62 (12.81)	53 (10.95)	131 (27.07)	49 (10.12)	29 (5.99)	20 (4.13)	
Paraguay	96 (23.02)	45 (10.79)	70 (16.79)	125 (29.98)	39 (9.35)	17 (4.08)	25 (6)	
Peru	48 (14.91)	26 (8.07)	39 (12.11)	98 (30.43)	53 (16.46)	31 (9.63)	27 (8.39)	
Uruguay	78 (19.9)	48 (12.24)	51 (13.01)	106 (27.04)	44 (11.22)	30 (7.65)	35 (8.93)	
Venezuela	69 (17.92)	48 (12.47)	52 (13.51)	96 (24.94)	37 (9.61)	33 (8.57)	50 (12.99)	

*ξ^2^: Epsilon squared (non-parametric effect size).*

## Discussion

The study was conducted during the second half of 2021, when Latin American countries were in the midst of the vaccination process against COVID-19, but there was still an important percentage of the population that refused to be vaccinated. In this sense, we sought to provide a quick overview of the variations in conspiracy beliefs about COVID-19 vaccines, which have proliferated rapidly during the pandemic, according to some sociodemographic variables in 13 Latin American countries. Thus, the findings could provide information to support prevention and treatment efforts during the COVID-19 pandemic.

First, in most countries, women have the highest support for conspiracy beliefs against a COVID-19 vaccine, which is consistent with other studies (Sallam et al., 2020a,2021a,2021b; Wang and Kim, 2021). This suggests that women tend to be more hesitant and fearful about COVID-19 vaccines (Lin et al., 2021; Murphy et al., 2021). This has been associated with men being less likely to believe in conspiratorial ideas about the origin of vaccines and viruses, because they mostly trust doctors, scientists and findings published in scientific journals, unlike women, who tended to trust information disseminated in social networks (Sallam et al., 2021a). Likewise, it was suggested that the lower perceived risk of COVID-19 by women could be associated with greater acceptance of conspiracy beliefs about the pandemic compared to men (Sallam et al., 2020b). Furthermore, the greater likelihood of women making decisions about children’s health would make them more likely to seek information about vaccines and be more exposed to anti-vaccine content (Smith and Graham, 2019). Similarly, women tend to score higher on disgust sensitivity, which is associated with greater vaccine hesitancy (Hornsey et al., 2018). However, in Uruguay and Venezuela, it is men who present greater support for conspiratorial beliefs, although these differences are insignificant. Studies suggest that higher levels of learned helplessness and uncertainty could explain this greater acceptance of conspiracy beliefs in males (Cassese et al., 2020). Other studies have suggested that beliefs in vaccine conspiracies did not differ by gender (Shapiro et al., 2016; Freeman et al., 2020b). Overall, then, the different results regarding COVID-19 vaccine conspiracy theories according to different genders seem to be mixed without a fully defined pattern (Tonkoviæ et al., 2021). In this regard, more research is needed on the role of gender in the acceptance or rejection of COVID-19 vaccine conspiracy beliefs considering other factors such as COVID-19 risk perception, health literacy, differential vulnerability to COVID-19, gender-associated comorbidity, and pre-existing doubts about vaccines in general (Khubchandani et al., 2021).

Another finding in this study was that less educated people are more likely to believe in conspiracies against COVID-19 vaccines, which is to be expected based on previous scientific literature (Allington et al., 2021a; Sallam et al., 2021a). This can be explained in part because less educated people tend to have less access to information about COVID-19 vaccines, which generates less certainty about their development, effectiveness and consequences (Omar and Hani, 2021). In this sense, it has been suggested that people with a university level education would be more likely to believe in the vaccine providing protection to those who receive it (Cordina and Lauri, 2021). However, in countries such as Chile and Ecuador, it was people with higher levels of education who were more in agreement with conspiracy beliefs about vaccines. It is possible that people with higher education consider that newer vaccines, such as those against COVID-19, may have more risk than older vaccines and therefore need more accurate information than less educated people (Smith, 2017). A study in Venezuela suggested that educational level was not a significant predictor in the acceptance of conspiracy theories (Andrade, 2021). Based on these results, governments need to strengthen and adapt communication strategies about the development and efficacy of vaccines, regardless of people’s educational level (French et al., 2020).

Some studies point out that, among demographic variables, age has shown the strongest association with vaccine hesitancy (Allington et al., 2021a); while others point out that it has little correlation with acceptance of conspiracy beliefs about the COVID-19 vaccine (Buturoiu et al., 2021; Jensen et al., 2021). Regarding age, the findings of the present study do not follow the same pattern. In fact, in Argentina, Colombia, and Paraguay, people older than 42 are the ones who agree more with conspiracy ideas; while in Cuba, Guatemala, Mexico, Uruguay, and Venezuela, people between 23 and 42 years old are the ones who support those beliefs the most. The latter is in agreement with studies which suggest that the adult population is particularly susceptible to believe in conspiracy ideas (Ðorđdević et al., 2021; Jensen et al., 2021). In the case of Bolivia, Chile, Peru and El Salvador, people under 23 years of age are those who agree most strongly with conspiracy beliefs. Recent studies assessing conspiracy beliefs related to COVID-19 support this finding (Romer and Jamieson, 2020; Allington et al., 2021b). This is associated with youth’s increased consumption of social media, which is the channel where vaccine-related conspiracy theories are most widely disseminated (Pew Research Center, 2021). The findings of the present study seem to suggest that people of all ages are vulnerable to conspiracy beliefs about COVID-19 vaccines. Therefore, it would be useful to further investigate the interactions between age and conspiracy beliefs to design solutions against misinformation among people of all ages.

Similarly, in countries such as Chile and Cuba, it was reported that people who use Facebook or social networks as sources of information about the vaccine and COVID-19 have a higher degree of agreement with conspiracy beliefs about vaccines. This is expected since people who tend to believe more in conspiracies and reject vaccines get more information from social networks and not from health professionals or verified health websites (Danielson et al., 2019). Moreover, about 52% of people who use the Internet consider it a reliable means of obtaining health information (Kata, 2010). The novelty of COVID-19 has led to the rapid spread of false news about the origin of the disease and its treatment. This type of information can confuse the population and generate a danger to their health, as is the case of news about the non-existence of the virus or that vaccines contain a microchip to control people (Ortiz-Sánchez et al., 2020). In the case of Chile, the finding is to be expected since Chile is one of the Latin American countries with the highest participation in the #yonomevacuno trend, where users expressed a diversity of opinions about the vaccine, the vaccination process or the COVID-19 pandemic (Herrera-Peco et al., 2021). Regarding Cuba, the finding is important considering that 7.1 million people (63%) have access to the Internet and 6.27 million (55%) are active in social networks (Alemañy-Castilla, 2020). Thus, the efforts of health professionals, health organizations, and social networks should be united to prevent the spread of false information (Ortiz-Sánchez et al., 2020). However, in most countries participants indicated that their main source of information about COVID-19 vaccines was family and friends. While there are efforts to discredit conspiracy theories or persuade people who believe in them (Earnshaw et al., 2020), this finding could suggest a need for developing complementary intervention strategies. Thus, for example, when these close people (friends or family members) convey the idea that getting vaccinated is a behavior that should be performed, conspiracy beliefs seem to stop predicting vaccination intentions (Earnshaw et al., 2020). This is important, even more so if one takes into consideration that attempts to influence people who believe in conspiracy ideas, based on communication coming from authorities, have failed (Lamberty and Imhoff, 2018). Thus, personalized health communication and coming from family and friends might be more successful (Sassenrath et al., 2018). Finally, less reliance on obtaining information from official sources of information may put people at risk of contracting the disease. This form of “system avoidance” could therefore have negative and paradoxical implications for individuals, and even increase susceptibility to disease in some social groups.

The analysis of the responses to each of the ECCV-COVID questions shows that, in general, the countries evaluated are mostly in some degree of disagreement or indecision with respect to the conspiratorial beliefs about the COVID-19 vaccines. However, there are also a number of people who support the conspiracy theories surrounding vaccination against COVID-19. For example, when adding up the positive responses (somewhat agree, agree, strongly agree) to question 1 alone, the results range from 24% in Chile and 25% in Cuba to 40% in Peru and 45% in Guatemala. In part, these differences can be explained on the basis of the construct level theory, which indicates that different beliefs can be interpreted differently and can also generate different degrees of impact on people. The different interpretations will depend on the psychological distance of the cognitive objects perceived by people. In this sense, when people perceive that the psychological distance between the belief and their behavior is large, then the belief has a smaller impact on their behavior (Trope and Liberman, 2010). In the present study, it appeared that conspiracy beliefs about the COVID-19 vaccine and vaccine-related knowledge were closer to the target behavior of the population in the Latin American countries evaluated (referring to the COVID-19 vaccination that was already in process) at the psychological level.

Another possible explanation for the observed differences could be associated with the political domain, which is an important area where conspiracy beliefs in general play a prominent role (Imhoff and Lamberty, 2018). Thus, for example, it has been suggested that conspiracy theories are closely related to the discourses of populist political leaders who tend to use conspiracy theories for strategic political management purposes (Bergmann, 2018). Likewise, other studies have reported a linear relationship between self-reported political orientation and the acceptance of conspiracy beliefs (Dieguez et al., 2015; Imhoff and Lamberty, 2018), suggesting that the presence of conspiracy beliefs is less common in people with a left-wing political orientation compared to those with a right-wing political orientation (Miller et al., 2016; Jost et al., 2018; Van der Linden et al., 2021). For example, in the case of Chile, since 1993 there has been a significant increase in people who identify with a left-wing orientation and a decrease in those identified with right-wing, center and center-right political orientations (Titelman, 2019). The increase in identification with left-wing politics has been reflected in the demand for economic, health and education changes which have occurred since 2019, which led to the installation of a new constitution as a path to a new society of rights. In the case of Peru, with a greater acceptance of conspiracy beliefs, it has been suggested that there is no political party system that allows people to identify the values that are associated with one political stance or another (Silva, 2018). In this sense, it has been indicated that in Peru there is a high perception of transgression of norms in society and a perceived lack of legitimacy in official institutions, such as those referring to the health system, which paints a picture of a weak and fragile normative system (Janos et al., 2018). Negatively perceived normative systems are characteristic of societies where corruption and transgression are recurrent practices (Beramendi and Zubieta, 2013) and considered normal or inevitable (Janos et al., 2018). In Peru, the vaccination program against COVID-19 was compromised in a political scandal linked to the application of vaccines to people outside the clinical trial being carried out in the country, an event called “Vacunagate” (Chauvin, 2021; Mayta-Tristán and Aparco, 2021). This has possibly helped to undermine confidence in vaccines and vaccination, leading to a greater proliferation of misinformation on the subject. The current study does not allow us to test this explanatory hypothesis, but future studies could focus on considering beliefs in specific conspiracy theories, such as those related to vaccination, as a product of latent political orientations.

Likewise, the health systems in place to face the pandemic vary among countries. For example, in Chile, there was an increase from 1,698 ICU beds in the National Health Services System before the pandemic to 38,571 total beds (2.2 per thousand inhabitants) (Arteaga Herrera, 2020) during the pandemic. In Cuba, at the beginning of the pandemic, 11 hospitals were designated for the care of COVID-19 patients, with an availability of 3,468 beds. As the number of patients increased, a greater number of hospitals, isolation and monitoring centers were set up, reaching a total of 20 institutions, and the availability of 7,471 beds, of which 477 were Intensive Care Units (ICUs). In Peru, at the beginning of the pandemic (April 2020), the country had only 133 ICU beds at the national level, which was increased during the pandemic to more than 2,000 beds (Ponce de León, 2021). However, the efforts of the Peruvian health system have not had adequate results, leading Peru to become one of the countries with the highest number of deaths in the Americas (Ramos, 2020). The inadequate management of the pandemic in different Latin American countries may have contributed to different levels of fear of the pandemic. It has been suggested that people with a greater fear of COVID-19 would direct their thoughts toward conspiracy theories about vaccines in order to diminish their fears by providing a justification for the difficulties (Stephens, 2020). The precariousness of health systems is not the only explanation for the acuteness of the pandemic in Latin America. There are other important factors that are associated with different responses to the pandemic and its outcomes in the different countries of the region, such as high levels of informality, unequal access to basic services, overcrowding and high population density, inadequate hospital infrastructure, inability of health systems to develop testing processes and early identification of cases, or lack of political leadership (Ramos, 2020). Future studies could provide objective clarification of these possible explanations.

Similarly, it is noteworthy that countries with a lower acceptance of conspiracy beliefs about vaccines against COVID-19, such as Chile or Cuba, are also those that show the greatest progress in the complete vaccination of the majority of their citizens at the time of the study (see Figure 2), while participants in Guatemala seem to have problems of confidence in vaccination, with only 17% of the population fully vaccinated at the time of data collection. In this sense, it appears that confidence in vaccines may also be a factor explaining the differences in the vaccination coverage (Jovančević and Milićević, 2020). It has been suggested that lower levels of general trust predict greater acceptance of conspiracy beliefs (Wood and Douglas, 2013). The spread of trust about COVID-19 vaccines depends on the content of vaccination messages and the medium from which they come. People have more trust and quickly adopt the behaviors of those closest to them. Thus, information about COVID-19 vaccines from a family member may be more effective than information from an outsider (Anderson et al., 2020). However, it has also been reported that reliance on information provided by experts would affect safety behavior regarding COVID-19 vaccines. This could be observed, for example, in the case of Cuba, where people presented the highest levels of satisfaction and trust with the information on COVID-19 provided by health experts (Meda-Lara et al., 2021). In the case of Chile, the low acceptance of conspiracy beliefs about COVID-19 vaccines was likely related to the fact that only 23% of the population completely refused to be vaccinated (Cerda and García, 2021). Chile, together with Brazil, had the highest acceptance rates compared to other Latin American countries (Rosiello et al., 2021).

However, if we observe the percentages of acceptance of conspiracy beliefs in countries such as Peru, El Salvador or Uruguay, the association with vaccination rates is not entirely evident. Despite this, in Peru, the lack of trust in scientific information on COVID-19 and vaccines has fostered conspiracy ideas in different scenarios. For example, a group of people kidnaped workers performing maintenance on 5G cell phone antennas, based on the idea that they spread the SARS-CoV-2 virus (Vega-Dienstmaier, 2020). On the political side, Peruvian congressmen requested the creation of a commission that would evaluate the effects of chlorine dioxide in the treatment of COVID-19, for which they invited advocates of this product to present their ideas (Mostajo-Radji, 2021). It appears that the association is not fully defined and it is possible that other variables, such as accessibility, fear of adverse reactions, safety concerns and lack of motivation, may explain these differences (Sallam et al., 2021a). Still, the possible association between conspiracy beliefs about vaccines and vaccination rates should alert country health authorities and the various media to the negative effects of misinformation dissemination.

Misinformation associated with, for example, the death of children after receiving the COVID-19 vaccine in several countries have circulated widely; one such story that was spread on Facebook indicated the death of seven children after receiving the COVID-19 vaccine in Senegal (Islam et al., 2021). This has also been observed with conspiracy beliefs referring to other vaccines, such as those developed against mumps, measles, and rubella, which are erroneously thought to cause autism in children and autoimmune disorders in adolescents (Maglione et al., 2014). In the present study, among the different conspiracy beliefs showing agreement or disagreement, the one referring to “Vaccinating children against COVID-19 is harmful and this fact is hidden” shows the greatest difference. Similar results were observed previously (Romer and Jamieson, 2020; Yang et al., 2021). It has even been suggested that while about 92% of the world’s population believes that vaccines are important for children, there is also a large variation in support for this belief in some countries, ranging from 76% in France to 98% in India and Mexico; however, the causes of these variations are not entirely clear (Vanderslott et al., 2019). In addition, believing in conspiracies against vaccines, regarding their undisclosed harmful health effects, was related to lower willingness to vaccinate children (Jolley and Douglas, 2014). Later, direct arguments against conspiracy beliefs were shown to increase intentions to vaccinate a child when these arguments were presented prior to the emergence of conspiracy theories (Jolley and Douglas, 2017). However, once conspiracy theories became established, it was more difficult to correct them with arguments against these types of beliefs (Douglas, 2021). While the rates of hospitalization and death from COVID-19 in children are significantly lower than in adults, it is important for children to be vaccinated against the disease as well. However, having some degree of agreement with beliefs about the negative consequences of vaccination in children could affect their health. Although the priority for vaccination is high-risk groups in the adult population, it has been recommended that children at higher risk of severe and fatal disease should be vaccinated first, and then vaccination should be extended to other groups of children (Wong et al., 2021). Differences in the degrees of agreement or disagreement about erroneous beliefs about vaccination in children may be related to a lack of confidence and lack of knowledge about the importance of vaccination (Benin et al., 2006).

The study has some limitations. First, although the highest percentage of responses, in most countries, are in low response alternatives (1 and 4), it is recognized that the findings of the study may not be generalized to all populations in the countries evaluated, since an online form and non-probability convenience sampling were used. This method implied that all participants were volunteers and felt motivated to participate (Simione et al., 2021). However, this method was the only feasible one at the time of data collection, when most of the population in all participating countries had limited social interactions. Similarly, due to the type of sampling, the participants were mostly women and university-educated, which led to the presence of a sampling bias. Therefore, subsequent studies should have more homogeneous samples in each of the gender, age and educational level groups. Likewise, the use of a self-report questionnaire to assess conspiracy beliefs could also generate a social desirability bias. Furthermore, although the study was cross-sectional, the sample size in each country was relatively small compared to the total population. Given that this is a cross-sectional study, the present data do not allow us to draw conclusions about the variability of conspiracy beliefs throughout the pandemic as vaccination processes progress across countries. Thus, longitudinal studies are needed to detect any variation involved with conspiracy beliefs (Winter et al., 2021). For example, many conspiracy beliefs and misinformation have been debunked by international health agencies and, therefore, it is not known whether corrected information has led to changes in people’s original perceptions of vaccines (Islam et al., 2021). In addition, as scientific evidence on COVID-19 has advanced, information about vaccines has also changed and, therefore, some beliefs have also changed. Due to its exploratory and introductory nature, this study did not consider additional analyses on other sociodemographic or psychosocial variables that may contribute to the acceptance of conspiracy beliefs about COVID-19 vaccines. Thus, future studies may decide to address this limitation. Finally, it is possible that this study did not cover all the conspiracy beliefs circulating about COVID-19 vaccines. Therefore, the beliefs assessed may have underestimated the true prevalence.

The large amount of misinformation about COVID-19 vaccines currently circulating negatively impacts the vaccination process. The circulation of this type of information can be misinterpreted as credible information (Bontcheva et al., 2020). In this context, it is important to consider that the dissemination of misinformation, the increase of multimedia information manipulated by artificial intelligence, and the appearance of different harmful content issued by media and individuals (including health professionals) are some of the dangers to public health that people can find on social networks (Ferrara et al., 2020). Thus, it is important to also have collective immunity against misinformation and conspiracy beliefs to ensure collective immunity against COVID-19 (World Health Organization, 2020). This preliminary study suggests that, in most countries, women, people with a lower educational level and those who receive information about the vaccine and COVID-19 from family and friends are generally more supportive of conspiracy ideas against COVID-19 vaccines. In the case of age, the results are very mixed. Likewise, the belief referring to “Vaccinating children against COVID-19 is harmful and this fact is hidden” is the one that shows the greatest difference in agreement or disagreement between countries.

Despite the limitations, the findings in this study have important implications, some of which have already been suggested above. Thus, groups of people at increased risk for conspiracy beliefs about COVID-19 vaccines could be identified, in addition to preventing the development of new conspiracy beliefs and dispelling existing beliefs with the goal of promoting intervention strategies against COVID-19. Risk communication and community engagement should be emphasized to track and identify misinformation about vaccines as a way to address these concerns with evidence-based information and ‘immunize’ people against misinformation (Bontcheva et al., 2020). On the other hand, although there is scant information on cultural differences in COVID-19 vaccine conspiracy beliefs, that possible cultural differences are attributable to variations in the levels of uncertainty and fear experienced across cultures (van Prooijen and Douglas, 2017). Regarding the latter, a recent study concluded that there are differences in levels of fear of COVID-19 in Latin American countries (Caycho-Rodríguez et al., 2021). Furthermore, cultural differences in susceptibility to conspiracy beliefs are related to variations in trust, particularly in contexts of inequality where there is a variable distance between power elites and the masses, as occurs in many Latin American countries (van Prooijen and Van Vugt, 2018).

## Data Availability Statement

The original contributions presented in the study are included in the article/Supplementary Material, further inquiries can be directed to the corresponding author/s.

## Ethics Statement

The studies involving human participants were reviewed and approved by Ethics Committee of the Universidad Privada del Norte in Peru (registration number: 20213002). The patients/participants provided their written informed consent to participate in this study.

## Author Contributions

TC-R and JV-L provided initial conception, organization, and main writing of the text. JV-L analyzed the data and prepared all figures and tables. LWV, PV, CC-L, MR-B, MW, CR-J, RP-C, MG, MC, PM, DP, RM-H, AS-P, ML, AB, DP-C, IEC-R, RC, BP, WA, and OP were involved in data collection for their respective countries and acted as consultants and contributors to research design, data analysis, and text writing. All authors read and approved the draft.

## Conflict of Interest

The authors declare that the research was conducted in the absence of any commercial or financial relationships that could be construed as a potential conflict of interest.

## Publisher’s Note

All claims expressed in this article are solely those of the authors and do not necessarily represent those of their affiliated organizations, or those of the publisher, the editors and the reviewers. Any product that may be evaluated in this article, or claim that may be made by its manufacturer, is not guaranteed or endorsed by the publisher.

## Appendix 1

### Vaccine Conspiracy Beliefs Scale-COVID-19 (VCBS-COVID-19)

#### Spanish Version

1. La información sobre la seguridad de las vacunas contra la COVID-19 a menudo se inventan. (*COVID-19 Vaccine safety data is often fabricated*)
2. Vacunar a los niños contra la COVID-19 es perjudicial y este hecho está ocultado. (*Vaccinating children against COVID-19 is harmful and this fact is covered up*)
3. Las empresas farmacéuticas ocultan los peligros de las vacunas contra la COVID-19. (*Pharmaceutical companies cover up the dangers of COVID-19 vaccines*)
4. Se engaña a las personas sobre la eficacia de las vacunas contra la COVID-19. (*People are deceived about COVID-19 vaccine efficacy*)
5. La información sobre la eficacia de las vacunas contra la COVID-19 a menudo se inventan. (*COVID-19 Vaccine efficacy data is often fabricated*)
6. Se engaña a las personas sobre la seguridad de las vacunas contra la COVID-19. (*People are deceived about COVID-19 vaccine safety*)
7. El gobierno está tratando de ocultar el vínculo entre las vacunas contra la COVID-19 y la aparición de otras enfermedades. (*The government is trying to hide the link between COVID-19 vaccines and the appearance of other diseases*)
