# Supplementary figures and images for: What Is the Support for Conspiracy Beliefs About COVID-19 Vaccines in Latin America? A Prospective Exploratory Study in 13 Countries
Source: Front Psychol. 2022 May 6;13:855713. doi: 10.3389/fpsyg.2022.855713 (PMC9120924; doi:10.3389/fpsyg.2022.855713)

**Supplementary material**

**Figura S1**

*Boxplot chart for outlier’s review*
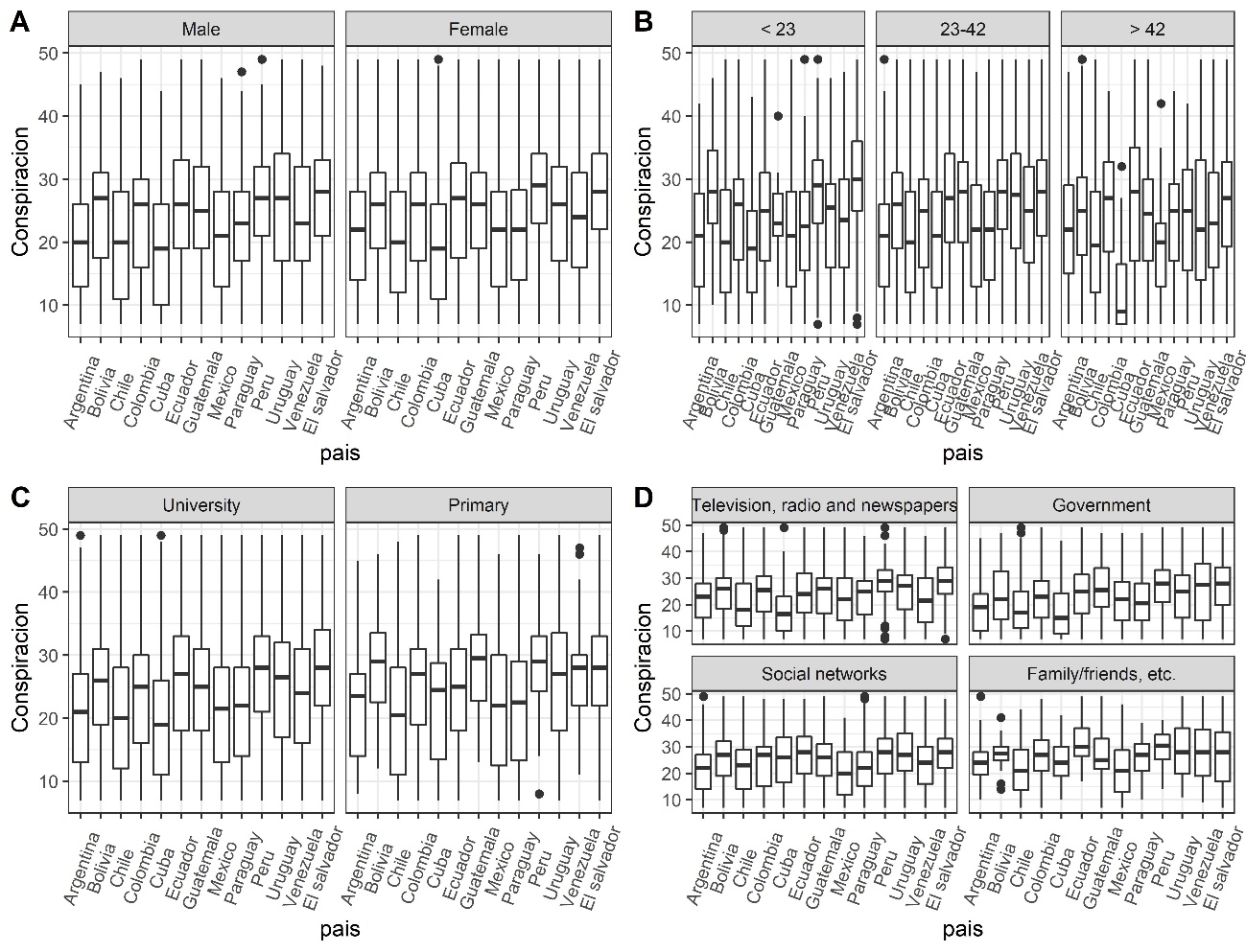

Supplement: Supplementary file 1 [file Table_1.DOCX]
